# Do pre-operative radiologic assessment predict postoperative outcomes in patients with insertional Achilles tendinopathy?: a retrospective database study

**DOI:** 10.1007/s00402-021-03897-x

**Published:** 2021-04-23

**Authors:** Sebastian Felix Baumbach, Hubert Hörterer, Sonja Oppelt, Ulrike Szeimies, Hans Polzer, Markus Walther

**Affiliations:** 1 Division for Foot and Ankle Surgery, Department of General, Trauma and Reconstructive Surgery, University Hospital, LMU Munich, Nussbaumstrasse 20, 80336 München, Germany; 2 Center for Foot and Ankle Surgery, Schön Klinik München Harlaching, Harlachinger Strasse 51, 81547 Munich, Germany; 3 Radiologie in München Harlaching, Grünwalder Str. 72, 81547 München, Germany; 4 Department of Orthopedics and Orthopedic Surgery, Julius- Maximilians-University, Brettreichstraße 11, 97074 Würzburg, Germany

**Keywords:** Achilles, Insertion, PROM, Imaging, Surgery

## Abstract

**Introduction:**

Diagnosis and treatment of insertional tendinopathy of the Achilles tendon (IAT) remains a challenge. The aim of this study was to assess the influence of pre-operative radiological pathologies on the patient-reported outcomes following open debridement of all pathologies for IAT.

**Materials and methods:**

In this IRB-approved retrospective correlation and comparative study, patients with pre-operative imaging were identified from the authors’ retrospective IAT database comprising of 118 patients. All were treated by a standardized surgical treatment strategy utilizing a midline, transachillary approach and debridement of all pathologies. A total of fifteen radiologic parameters were measured on radiographs (RX) and MRI. The patient-reported outcomes were assessed using the Victorian Institute of Sport Assessment-Achilles questionnaire (VISA-A-G) and the general health questionnaire SF-12 at a minimum follow-up of 12 months. The data are presented as mean ± SD (95% CI).

**Results:**

88 patients (74.6%) with an average age of 50 ± 12 (47–52) years were included. Radiographs were available in 68 patients and MRI in 53. The mean follow-up was 3.8 ± 1.9 (3.4–4.3) years. The overall VISA-A-G was 81 ± 22 (77–86), the SF-12 PCS 54 ± 7 (52–55), and the SF-12 MCS 52 ± 9 (50–54) points. None of the assessed radiological parameters had a significant influence on the patient-reported outcome following surgical treatment for IAT.

**Conclusion:**

In this retrospective correlation study, no significant association was found between preoperative radiographic and MRI radiologic parameters for IAT and postoperative patient-reported outcomes (VISA-A-G and SF-12).

## Introduction

Although a common pathology, the diagnosis, and treatment of insertional tendinopathy of the Achilles tendon (IAT) is difficult in many ways. Historically, researchers have tried to identify radiographic parameters predictive for IAT. These include the Fowler & Philip angle [1], Parallel pitch lines [2], Calcaneal pitch angle [3] and the Chauveaux–Liet angle [4]. In more recent years, the diagnostics of IAT have been extended by MRI. In 2011, van Dijk et al. classified IAT per its underlying pathologies [5]. The insertion of the Achilles tendon can be subdivided into three compartments, the retro-achillary-, intra-achillary- and pre-achillary compartment. Per these compartments, the following pathologies can be subdivided: retrocalcaneal bursitis, Achilles tendon degeneration including partial ruptures, calcifications in the tendon, dorsal bone spurs, and superficial calcaneal bursitis [6]. But up to now, it remains unclear which of these pathologies (radiographic and MRI) are causative of the pain in IAT, as these changes have also been observed to a considerable percentage in asymptomatic populations [7]. Some authors have even challenged the necessity of preoperative MRI at all [8].

Following the diagnosis of IAT and identification of the radiologic abnormalities, non-operative treatment should be initiated over a period of 3–6 months [9, 10]. In the case of failed non-operative treatment, surgery can be considered [11]. As it remains unknown which of the pathologies identified by radiographs and MRI are responsible for painful IAT, open debridement of all pathologies is regularly performed [9, 12–14]. Open debridement is performed through extensile approaches and often necessitates a (partial) detachment of the Achilles tendon from its calcaneal insertion. Although this invasive approach results in good to excellent patient satisfaction rates in over 80% of patients [15–17], about 20% of patients remain impaired and complication rates vary between 7 and 30% [12, 14, 18, 19].

One of the most important responsibilities of any surgeon is to give the patient a realistic expectation on the outcome of a surgical intervention [20]. Previous studies have either compared radiographic parameters between symptomatic and asymptomatic patients [7, 21–27] or have simply investigated the outcome of surgically treated IAT [14, 15, 19]. But to the authors’ best knowledge, no study has yet investigated whether any of the above-mentioned radiologic parameters (radiographic and MRI) are predictive of the patient-reported outcomes following surgical debridement in IAT. This would enable the surgeon to give the patient a realistic expectation on the expected outcome. Furthermore, it might help to develop new treatment approaches for those patients with residual symptoms.

Our hypothesis was that certain pre-operative radiological pathologies (radiographic and MRI) in IAT predispose impaired patient-reported outcomes following surgery. The aim of this study was therefore, to assess the influence of any pre-operative radiological pathology (radiographic and MRI) on the patient-reported outcomes following surgical treatment.

## Materials and methods

The herein presented retrospective correlation and comparative study was approved by the local ethics committee (LMU # 17-804).

**Fig. 1:**
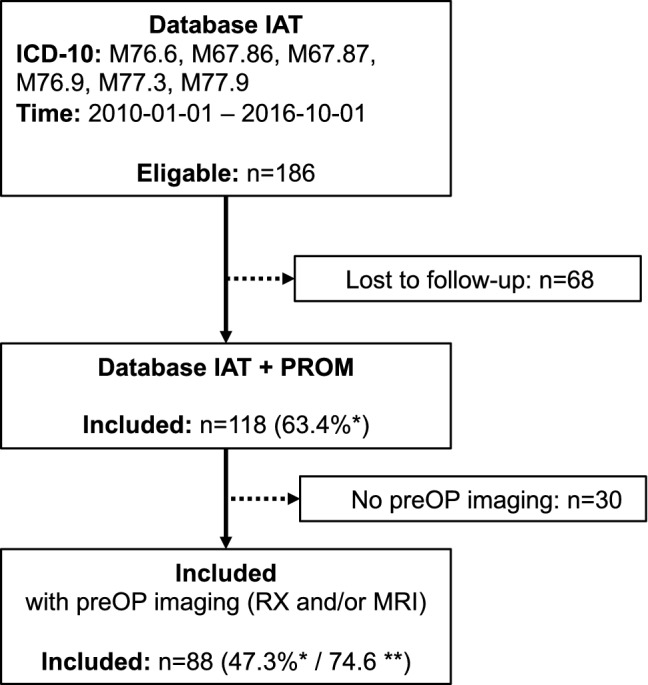
Flow-chart depicting patient selection. *IAT* Insertional Achilles tendinopathy; *n* Number of patients; *PROM* Patient-reported outcome measures; *PreOP* pre-operative; *RX* Plain radiographs; *MRI* Magnetic Resonance Imaging. *Percentage calculated to the IAT Database (*n* = 186); **Percentage calculated to the IAT + PROM Database (*n* = 118)

The patient selection is depicted in Fig. 1. It was based on the authors’ retrospective database for insertional tendinopathy of the Achilles tendon, which was descripted in detail previously [12]. In summary, the database comprises 118 patients who were treated surgically for an IAT between 01/2010 and 10/2016 with a PROM follow-up of at least 12 months. Revision cases were excluded. Any patient with pre-operative imaging (RX or MRI) was eligible. Overall, 88 patients (74.6%) were included in further analysis.

## Surgical treatment strategy

As outlined previously [12], all patients were treated surgically following at least 6 months with failed non-operative treatment. All patients were treated by a standardized surgical treatment strategy. In prone position, a midline, transachillary approach was utilized. All previously detected pathologies on radiographs and MRI were addressed. In case the Achilles tendon was detached more than 50% of its insertion, it was reattached using an anchor. In case of detachment < 50%, the vertical tendon split was sutured. A walker was applied for 6 weeks in neutral position if the Achilles tendon was not reattached or in equinus with stepwise reduction to neutral position over a period of 8 weeks, if the Achilles tendon was reattached with anchor(s). Following a two-week period of 10 kg partial weight-bearing, patients were allowed to progress to full weight-bearing.

## Imaging assessment

**Fig. 2:**
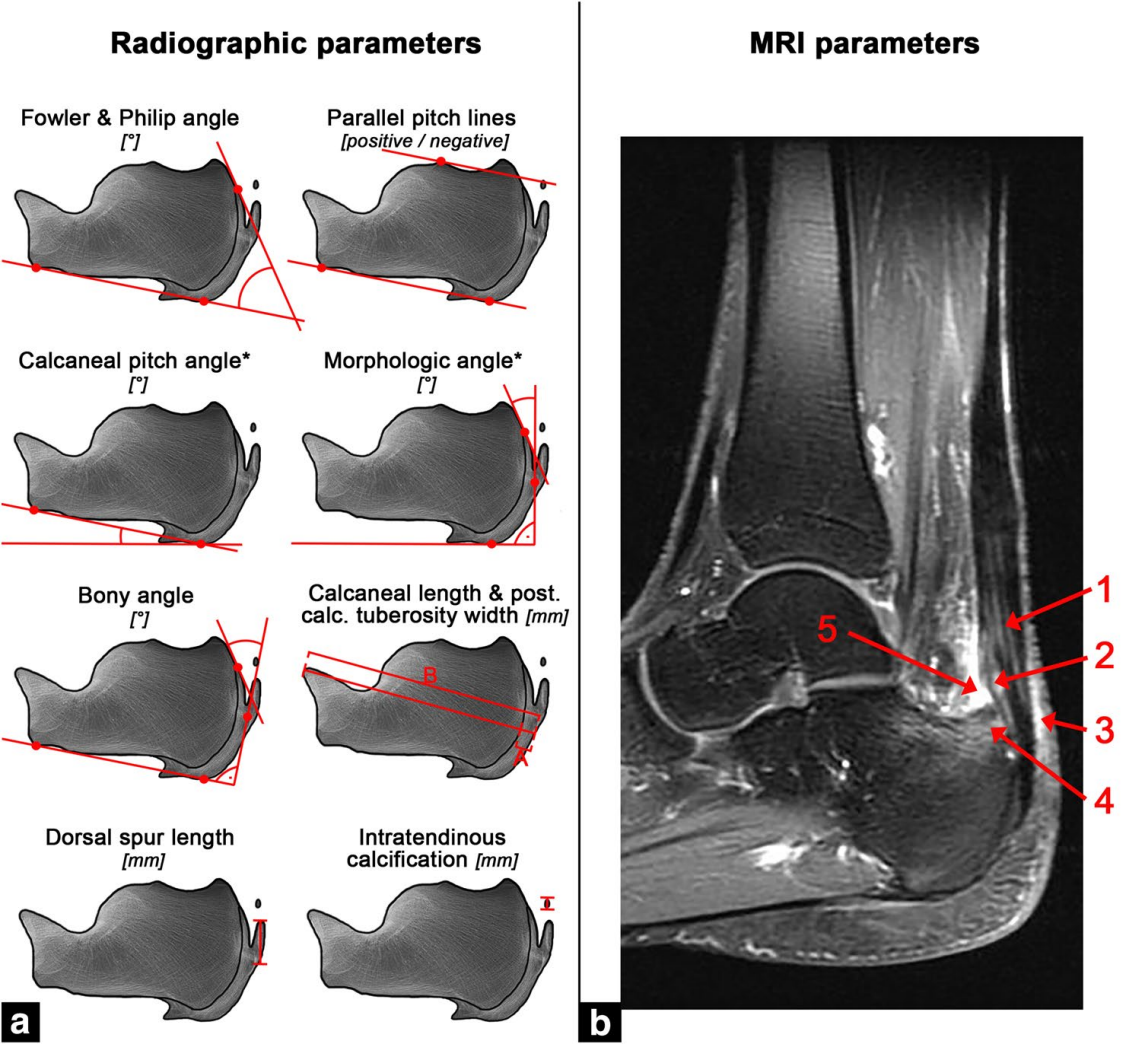
Illustration of the radiologic parameters assessed on lateral radiographs of the foot (A) and MRI (B). [°]: Degrees; *Could only be assessed on weight-bearing radiographs; [mm]: Millimeters; 90° angle. **A** Dorsal cortical width; **B** Maximum calcaneal length; *Post. calc. tuberosity width* Posterior calcaneal tuberosity width

The pre-operative RX and/or MRI were assessed for any common pathology associated to IAT (Fig. 2). The following parameters were evaluated on lateral radiographs of the foot (Fig. 2a). The Fowler & Philip angle is the angle between the baseline and a line tangential to the postero-superior calcaneal prominence (Haglund’s exostosis) with a normal range of 44–69°. Angles greater than 75° are considered to cause symptoms [1, 22]. The Parallel pitch line, as described by Pavlov et al., is a cranially parallel shift of the baseline to the most cranial point of the subtalar joint [2]. If the posterosuperior calcaneal prominence (Haglund’s exostosis) projects above this line, it is considered abnormal (positive). The Calcaneal pitch angle can only be assessed on weight-bearing radiographs and is the angle between the baseline and the ground [3]. Physiological angles range between 15–17° with most authors considering values between 20–30° intermediate, and angles above 30° as pathological [21, 22]. The Chauveaux–Liet angle is the difference between the Calcaneal pitch angle and Morphologic angle, which represents the angle between the ground and a line tangential to the posterosuperior calcaneal prominence (Haglund’s exostosis) on weight-bearing lateral radiographs [4]. Values of the Chauveaux–Liet angle greater than 12° are considered abnormal. As an alternative to the Chauveaux–Liet angle in non-weight-bearing radiographs, the authors introduced the Bony angle [4]. It is formed between a line perpendicular to the baseline and a line tangential to the postero-superior calcaneal prominence (Haglund’s exostosis). The relative posterior calcaneal tuberosity width of the posterior calcaneal tuberosity was calculated as the percentage of the dorsal cortical width to the maximum calcaneal length [1]. Finally, the presence and maximum length of a dorsal spur and intratendinous calcifications were documented [7]. All radiographs were assessed by two independent fellowship-trained orthopedic surgeons with at least 5 years of experience in foot and ankle surgery (SFB, HH) and mean values calculated. In case of disagreement of > 5°, the measurements were conducted together until agreement was reached.

Where present, pre-operative MRIs were analyzed per the recommendations of van Dijk et al. (Fig. 2b) [5]. These were Achilles tendon degeneration (none/minor/considerable), partial ruptures (none/small/medium/large), superficial calcaneal bursitis (bivariant), retrocalcaneal bursitis (bivariant), and the posterosuperior calcaneal prominence (Haglund’s exostosis; none/yes without bone marrow edema (BME)/ with BME). All MRIs were rated by a senior radiologist specialized in foot and ankle imaging (US).

## Outcome assessment

The outcomes were assessed with the latest follow-up using validated patient-reported outcome scores (PROMs). The scores chosen were the functional outcome score Victorian Institute of Sport Assessment-Achilles questionnaire (VISA-A-G) [28] and the general health questionnaire SF-12 [28, 29]. The VISA-A-G is a functional outcome score specifically designed for chronic Achilles tendinopathy and ranges between 0–100 points. Scores above 90 points resemble full recovery [28]. The SF-12 is one of the most frequently used quality of live scores. Scores of 50 equal those of a health reference population [29]. Finally, the patients were asked whether they were satisfied with the surgical outcome using a three-item Likert scale (not satisfied, intermediate, very satisfied).

## Statistics

The Shapiro–Wilk test revealed a normal distribution for scaled variables. Therefore, parametric testing was applied, and values were presented as mean ± SD (95% CI). Due to multiple testing, a Bonferroni correction was conducted, setting the alpha level to *p* < 0.004. Statistics applied were the chi-squared test, independent *t* test, ANOVA with Tukey post hoc, and Pearson correlation, where suitable.

## Results

The overall population was on average 50 ± 12 (95% CI: 47–52) years old, 34%/66% of patients were female/male. The left/right side were affected in 40%/60% of cases. Their mean BMI was 28 ± 5 (95% CI: 27–29), with an average ASA score of 1.6 ± 0.5 (95% CI: 1.5–1.7). The radiologic parameters per group (RX, MRI), as well as the group-specific demographics, are presented in Table 1. Overall, no significant group differences were found for the demographic variables or for the radiographic parameters between the cohorts RX + MRI and RX.

**Table 1:** Cohort specific deliberation of the demographic and imaging parameters

	RX (*n* = 68/WB *n* = 23)	MRI (*n* = 53)
*Demographics*		
Age	50 ± 12 (47–53)	51 ± 11 (48–53)
Sex [%female]	34%	34%
BMI	28 ± 4 (27–29)	28 ± 6 (26–29)
ASA	1.6 ± 0.5 (1.5–1.7)	1.6 ± 0.5 (1.5–1.8)
Side [%left]	43%	30%
*Radiographic parameters*		
Fowler & Philip angle [°]		
Overall	65 ± 8 (63–67)	–
Pathological [%]	10%	
Parallel pitch lines [% positive]	56%	–
Calcaneal pitch angle [°]*		
Overall	22 ± 4 (20–24)	–
Increased [%]**	61%	
Morphologic angle [°]*^,†^	7 ± 8 (3–10)	–
Chauveaux–Liet angle [°]*		
Overall	15 ± 9 (11–19)	–
Pathological [%]	61%	
Bony angle^†^ [°]	27 ± 8 (25–29)	–
Posterior calcaneal tuberosity width^†^ [mm]	8 ± 2 (7–8)	–
Calcaneal length^†^ [mm]	90 ± 7 (88–92)	–
Relative posterior calcaneal tuberosity width [%]	9 ± 2 (8–9)	–
Dorsal spur [mm]	*n* = 38 10 ± 6 (8–12)	–
Intratendinous calcifications [mm]	*n* = 19 6 ± 4 (4–8)	–
*MRI parameters*		
Tendon degeneration [% yes]	–	81%
Partial tendon ruptures [% yes]	–	60%
Retrocalcaneal bursitis [% yes]	–	64%
Postero–superior calcaneal prominence (Haglund’s exostosis) [% yes]	–	74%
Superficial calcaneal bursitis [% yes]	–	15%

If not stated differently, values are presented as mean ± SD (95% CI)

*WB* Weightbearing

*Assessed on weight-bearing radiographs

**20° cut-off was chosen, as only one patient had an angle above 30°

^†^No published reference values

The mean follow-up period after surgery was 3.8 ± 1.9 (95% CI: 3.4–4.3) years. At that time point, the overall patient-reported outcome for the VISA-A-G was 81 ± 22 (95% CI: 77–86). The SF-12 revealed a PCS of 54 ± 7 (95% CI: 52–55) and an MCS of 52 ± 9 (95% CI: 50–54). Overall, patient satisfaction was rated as not satisfied in 5%, intermediate in 18%, and very satisfied in 77%. In the first step, we analyzed whether any of the assessed demographic or radiologic parameters significantly influenced the PROMs assessed (Table 2). Although three parameters (BMI, Calcaneal pitch angle total value, and percent pathological) showed significant results for individual PROMs, these findings were not consistent throughout the outcome parameters assessed. In a second step, the VISA-A-G was grouped per full recovery (< 90 points/ ≥ 90 points), and the analysis was repeated. None of the assessed parameters differed significantly between patients with residual symptoms or fully recovered patients per the VISA-A-G score (< 90 points/ ≥ 90 points).

**Table 2:** Analysis of possible parameters affecting the patient-reported outcome

	VISA-A-G		SF-12 PCS	SF-12 MCS
	Total	Binary < 90 points: *n* = 41 ≥ 90 points: *n* = 47		
*Demographics*				
Age	0.142	0.107	0.656	0.383
Sex	0.635	0.362	0.883	0.413
BMI	0.967	0.740	**0.002**	0.326
ASA**	0.306	0.278	0.078	0.721
Side	0.297	0.403	0.888	0.807
*Radiographic parameters*				
Fowler & Philip angle				
Total value	0.806	0.487	0.995	0.934
Pathological (no/yes)	0.894	0.751	0.387	0.324
Parallel pitch lines	0.676	0.446	0.967	0.103
Calcaneal pitch angle*				
Total value	0.023	0.068	**0.004**	0.647
Pathological (no/yes)***	**0.002**	0.526	0.018	0.475
Chauveaux–Liet angle*				
Total value	0.697	0.381	0.835	0.169
Pathological (no/yes)	0.982	0.675	0.782	0.465
Bony angle^†^	0.314	0.289	0.045	0.607
Relative posterior calcaneal tuberosity width^†^	0.952	0.345	0.030	0.698
Dorsal spur [binary]	0.279	0.446	0.070	0.126
Dorsal spur [mm]	0.009	0.038	0.059	0.079
Intratendinous calcifications [binary]	0.048	0.133	0.213	0.352
Intratendinous calcifications [mm]	0.615	0.953	0.864	0.798
*MRI parameters*				
Tendon degeneration	0.837	0.876	0.191	0.055
Partial tendon ruptures	0.113	0.711	0.380	0.943
Retrocalcaneal bursitis	0.569	0.820	0.155	0.057
Postero-superior calcaneal prominence (Haglund’s exostosis)	0.634	0.887	0.817	0.391
Superficial calcaneal bursitis	0.216	0.288	0.606	0.580

Values presented are *p*-values

*Assessed on weight-bearing radiographs

**As only one patient had an ASA 3 and non an ASA 4 score, an independent sample *t* test was performed

*** 20° cut-off was chosen, as only one patient had an angle above 30°

^†^No published reference values

## Discussion

A considerable number of patients suffer from residual symptoms following open debridement of all pathologies in IAT. We had hypothesized that pre-operative radiological pathologies (radiographic and MIR) in IAT could be predictive for a poor patient-reported outcome. Based on 88 patients, 15 radiologic parameters and two validated patient-reported outcome measures were assessed at a mean follow-up of 3.8 ± 1.9 (95% CI: 3.4–4.3) years. No isolated parameter could be identified to correlate and predict impaired patient-reported outcome following surgical debridement of all pathologies in IAT.

The study aimed to identify radiologic parameters predictive of the surgical outcome in IAT. Previous studies have predominantly tried to define parameters differentiating symptomatic from asymptomatic patients using either radiographic or MRI parameters [7, 21–26]. Most comparative studies facilitated radiographic parameters [7, 21, 24–26]. Radiographic measurements are well established in the literature and date back as far as to 1945 for the Fowler & Philip angle [1]. But their significance remains debatable. Often, the initial descriptor of a parameter reported promising results (Fowler & Philip angle [1]; Parallel pitch line [2]; Calcaneal pitch angle [3]; Chauveaux–Liet angle [4]. But the vast majority of follow-up studies could not confirm their significance (Fowler & Philip angle [1]: [2, 25]; Parallel pitch line [2]: [21, 26]; Calcaneal pitch angle [3]: [2, 21, 25]; Chauveaux–Liet angle [4]: [21]). The rational behind most of these parameters is the quantification of the retroachillary space, i.e., the space between the postero-superior osseous calcaneal boarder and the Achilles tendon. A decrease in the retroachillary space has been hypothesized to increase the pressure on the Achilles tendon and thereby cause IAT. But there appears to be a great inter-individual heterogeneity for the assessed radiographic parameters, and so they are incapable of differentiating symptomatic from asymptomatic individuals.

As plain radiographic parameters have failed to reproducibly identify symptomatic patients, great expectations were placed on the MRI. Whereas radiographic parameters resemble indirect causes for IAT, MRI allows to directly visualize pathologies within and around the Achilles tendon [12]. However multiple studies were able to show, that abnormal MRI findings are also present in almost 33% of asymptomatic populations [30, 31]. This number is in line with histopathological studies [32]. Consequently, the value of preoperative MRI has been challenged [8]. Overall, none of the proposed parameters can diagnose with certainty the causative pathology in IAT. Therefore, any common radiologic parameter, radiographic as well as MRI, has been included in this study.

From a surgical perspective, we are in the dilemma of observing various pathologies on radiographs and MRI that could possibly be the source of pain in IAT. But we have no means of identifying those pathologies, that actually cause the pain in a particular patient. Due to this diagnostic uncertainty, the current operative treatment standard for IAT after failed non-operative therapy is the operative debridement of all pathologies detected on radiographs and MRI. This is most often done through a midline incision, transachillary approach. This extensile approach allows to address all pathologies with over 80% of patients reporting good to excellent results [16, 17]. These figures are comparable to the herein reported 77% of very satisfied patients. The VISA-A-G score (81 ± 22 (95% CI: 77–86) points) was slightly lower but comparable to previous studies using a similar approach. Hardy et al. reported a VISA-A of 92 ± 5.6 points and Miao et al. 87.9 ± 7.1 points [33, 34]. Despite these overall good results, 20% of patients are not fully satisfied with the surgical outcome and approximately 15% suffer complications [12]. The vast majority of complications are surgical-site infections, which are most likely related to the extensile surgical approach [12].

As outlined above, most previous studies using radiologic parameters have tried to differentiate symptomatic from asymptomatic patients [7, 21–27]. Furthermore, multiple studies have investigated the patient-reported outcome of surgically treated IAT [14, 15, 19]. But only very few studies have assessed the impact of radiologic parameters on the patient-rated outcome. Based on a retrospective chart review of 157 non-operatively treated IAT patients, Nicholson et al. found that patients with increased intramural tendon degeneration, classified per MRI, were unlikely to respond to non-operative treatment [35]. Hardy et al. planned the invasiveness of their surgical procedure on the extent of Achilles tendon degeneration based on pre-operative MRI. Consequently, it was unknown whether pre-operative radiologic parameters can predict the outcome of surgically treated IAT [33].

Knowing which radiologic parameters negatively influence the outcome could help to not only identify the most appropriate treatment strategy but would also enable any foot and ankle surgeon to give the patient a realistic outlook on the expected outcome [20]. A recent study on patients undergoing foot and ankle surgery just emphasized the importance of adequate pre-operative patient education. Based on 202 patients, MacMahon et al. showed that two-thirds of the patients had higher expectations regarding the outcome compared to their surgeon [20]. The herein presented study was the first to investigate the influence of pre-operative radiologic parameters on the patient-reported outcomes following surgical treatment for IAT. Despite the considerable number of patients and radiologic parameters assessed, no consistent correlation could be found between preoperative imaging and the patient-reported outcomes following surgery for IAT.

The study presented has several limitations that must be discussed. Most pronounced, the study design was retrospective. Although most studies comparing radiologic parameters in symptomatic to asymptomatic patients  have been retrospective, it is one of very few studies that not only assessed radiographic and MRI parameters but also assessed the outcome by standardized patient-reported outcome scores. Second, due to the retrospective design of the study, complete imaging sets (radiographs and MRI) were not available for all patients [7, 21, 23, 24, 26, 33, 36]. In addition, the MRI protocol was not standardized. MRI imaging was performed on 1.5 and 3 T machines with and without contrast agent. Therefore, the study might have been underpowered. But again, the number of patients included compares favorably to most previous studies assessing patient-reported outcome scores in IAT [23, 26, 33, 36]. The next limitation could be the extensile surgical treatment approach. Although a uniform, standardized treatment protocol could be seen as a strength, it does limit its significance. Future studies should apply different surgical treatment strategies in patients with comparable pathologies. This might help to identify pathologies that do not necessitate surgical treatment, which again reduces the invasiveness and therefore possibly the complication rates.

## Conclusion

Pre-operative radiographic and MRI imaging are the working-horse in the identification of pathologies associated to IAT. In this retrospective correlation and comparative study, pre-operative imaging cannot predict the outcome in surgically treated IAT. It can be hypothesized, that other factors, such as genetics or the regenerative capacity of the Achilles tendon (insertion), are essential for the outcome of surgical treatment. Future prospective and powered studies are needed to assess better predictive factors of improved outcomes in IAT patients.
